# Prokaryotic Phylogenies Inferred from Whole-Genome Sequence and Annotation Data

**DOI:** 10.1155/2013/409062

**Published:** 2013-08-29

**Authors:** Wei Du, Zhongbo Cao, Yan Wang, Ying Sun, Enrico Blanzieri, Yanchun Liang

**Affiliations:** ^1^Key Laboratory of Symbol Computation and Knowledge Engineering of the Ministry of Education, College of Computer Science and Technology, Jilin University, Changchun 130012, China; ^2^College of Chemistry, Jilin University, Changchun 130012, China; ^3^Department of Information and Communication Technology, University of Trento, 38050 Povo, Italy

## Abstract

Phylogenetic trees are used to represent the evolutionary relationship among various groups of species. In this paper, a novel method for inferring prokaryotic phylogenies using multiple genomic information is proposed. The method is called CGCPhy and based on the distance matrix of orthologous gene clusters between whole-genome pairs. CGCPhy comprises four main steps. First, orthologous genes are determined by sequence similarity, genomic function, and genomic structure information. Second, genes involving potential HGT events are eliminated, since such genes are considered to be the highly conserved genes across different species and the genes located on fragments with abnormal genome barcode. Third, we calculate the distance of the orthologous gene clusters between each genome pair in terms of the number of orthologous genes in conserved clusters. Finally, the neighbor-joining method is employed to construct phylogenetic trees across different species. CGCPhy has been examined on different datasets from 617 complete single-chromosome prokaryotic genomes and achieved applicative accuracies on different species sets in agreement with Bergey's taxonomy in quartet topologies. Simulation results show that CGCPhy achieves high average accuracy and has a low standard deviation on different datasets, so it has an applicative potential for phylogenetic analysis.

## 1. Introduction

There are about 10 to 500 thousand species of prokaryotes living on the Earth today [1]. Prokaryotes have a more complicated evolutionary relationship than Eukaryotes through their long existence. Owing to evolving in different environments, the prokaryotes have considerable diversity in both genetical and physical processes to adapt to different conditions. Phylogenies are used to represent the evolutionary relationship among various groups of species. So, studying the phylogenies of different prokaryotes can help us understand the similarities and differences in genotype and phenotype among them. Woese and Fox first proposed molecular phylogeny of prokaryotes using the small subunit ribosomal RNA (SSU rRNA) universal distribution [2]. Since then, rRNAs were commonly recommended as the molecular standard for reconstructing phylogenies [3, 4]. Phylogeny of prokaryotes inferred by rRNAs or genes has been immensely successful. However, there are some problems in this kind of methods. The phylogenetic trees inferred from single rRNAs or genes may have many conflicts because of the various biological phenomena [5], such as horizontal gene transfer (HGT) [6–8], hybridization, lineage sorting, paralogous genes [9], and pseudogenes [10, 11].

With the development of high-throughput sequencing technology, more and more genomic sequences of organisms have been determined. With the increasing availability of genome information, it became possible to infer phylogenies by computational methods. In the past decade, many algorithms for phylogenies reconstruction have been proposed. Teichmann and Mitchison developed a method by using orthologous protein families [12]. Ge et al. built the phylogenetic tree using clusters of orthologous groups COG to represent major phylogenetic lineages of encoded protein [13]. Ciccarelli et al. presented an automatic procedure for reconstructing the phylogenetic tree with branch lengths comparable across all three domains which contain archaea, bacteria, and eukaryote [14]. Daubin et al. suggested a supertree method to build bacterial phylogenetic trees through considering core genes [15]. However, these methods only used one type of genomic information to infer phylogenetic tree and could not achieve very accurate results [5].

There are also many methods of phylogenetic analysis using whole-genome datasets. Snel et al. presented a phylogeny construction model based on a similarity between two species, which was defined as the ratio of the number of common genes to the total number of genes [16]. Henz et al. extended the model by using nucleotide segment pairs instead of genes [17]. Luo et al. suggested using overlapping gene information to infer the genome phylogenies [18]. Deeds et al. developed a method for inferring phylogenies by protein structural domain [19]. Wu et al. used the frequencies of nucleotide string to infer phylogenies [20]. Qi et al. and Xu and Hao proposed composition vector tree (CVTree), a method of inferring prokaryotic phylogenies by the oligopeptide content in the complete proteomes [21–23]. Gao et al. compared the results of CVTree with the bacteriologists' taxonomy [24]. Lin et al. developed a tool based on composite distance matrix to produce prokaryotic phylogenies [5].

**Figure 1:**
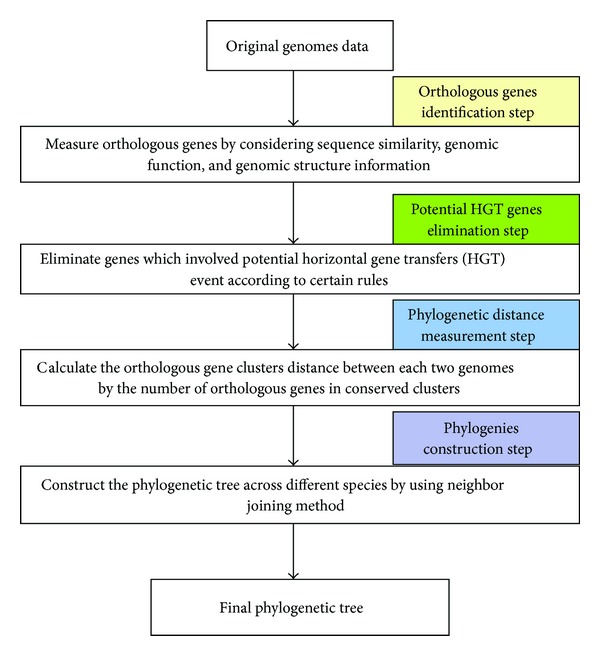
The pipeline of the CGCPhy.

Most of the methods presented above used several genome information, such as the sequence similarity, the genomic function, or the genomic structure. Moreover, these methods did not consider horizontal gene transfer (HGT) events and paralogous genes, and consequently they incorrectly classified some species on phylogenetic tree [25, 26]. In this paper, we propose a novel method called conserved gene cluster phylogenies (CGCPhy), which removes the genes potentially involved in HGT events, for inferring prokaryotic phylogenies. CGCPhy is based on the distance matrix of the orthologous gene clusters computed on whole genome and uses multiple genome information. The distance based on the orthologous gene clusters between two genomes is calculated by the number of orthologous genes in conserved clusters. The genes in an orthologous gene cluster are neighbour genes in a genome, and all of them have orthologous genes in the corresponding compared species. The pipeline of the method is shown in Figure 1.

In the first step, we compute orthologous genes by considering sequence similarity, genomic function, and genomic structure information. Sequence similarity is calculated by BLAST, whereas genomic function and genomic structure are estimated by COG and operon annotation, respectively. Consistently with other molecular phylogeny approaches, we suppose that the closer two species are in the phylogenetic tree, the more similar their genomic sequence, their function, and structure will appear. In the second step, the genes that are potentially involved in HGT events are eliminated according to certain criteria. In our work, two kinds of putative genes potentially involved in HGT events are considered. One kind contains the highly conserved genes across different species; take two strains from the same species as an example, almost all genes are highly conserved genes, and the other kind contains the genes located on fragments with abnormal genome barcode. After that, the distance matrix is calculated considering the number of orthologous genes in conserved clusters. Each element of the matrix is the conserved gene cluster distance (CGCD) that measures the conserved extent of orthologous gene clusters between two genomes. Finally, the neighbor-joining (NJ) [27, 28] distance-based method is employed to construct the phylogenetic tree by using the tool “Phylip” (available at: http://www.phylip.com/). CGCPhy is examined on different datasets from 645 prokaryotic genomes. In order to facilitate result comparison, the proposed method is firstly examined on the datasets defined for the evaluation of ComPhy [5]. In order to verify the effect of different methods further, we also validate these methods on random datasets which containa different number of species. The results of CGCPhy achieve higher accuracies than other existing methods both on fixed datasets and random datasets in agreement with Bergey's taxonomy [29] in quartet topologies. Simulation results show that the proposed method CGCPhy has consistently better performance on different datasets, so it has an applicative potential for phylogenetic analysis.

## 2. Materials and Methods

### 2.1. Data Preparation

The datasets of the 617 complete single-chromosome genomes and 28 multiple-chromosome prokaryotic genomes used to measure the performance of the methods in this paper were downloaded from the NCBI database (download date: June 1, 2013). Several kinds of genome information are used in our work, such as protein sequence information, location annotation, and COG function information [30]. The information is obtained from annotation files, which have been downloaded from NCBI. The operon data of each genome are obtained from DOOR [31]. The reference taxon data of the 645 prokaryotic genomes are defined by Bergey's code [29]. The Bergey's code is the abbreviation of the phylogenetic lineage in the Bergey's Manual of Systematic Bacteriology. For example, B12.2.3.1.3 is the abbreviation of the lineage Phylum XII (Proteobacteria), Class II (Betaproteobacteria), Order III (Methylophilales), Family I (Methylophilaceae), and Genus III (Methylovorus). In 2009, 2010, and 2012, the Bergey's Manual is updated gradually [32–34]. Due to the small part of species of these updated versions, we use Bergey's Manual of Systematic Bacteriology (release 5.0) [29] to measure the inferring performance of a method. However, the constructed phylogenetic trees are shown using the new version of Bergey's code.

### 2.2. Orthologous Genes Identification

In CGCPhy, the first step of phylogenetic inference is to determine orthologous genes used to measure the similarity between two species. Several methods have been proposed for identifying orthologous genes between species through sequence comparison, such as all-against-all BLAST [16]. Lin and coauthors [5] measured orthologous genes by the reciprocal best BLAST method and determined orthologous genes with *E*-value below 10^−3^ and sequence identity higher than 30%. Sequence information is extremely significant in the identification of the orthologous genes. However, inferring phylogenetic trees only by sequence similarity presents several problems. Horizontally transferred genes, paralogous genes, and pseudogenes may have high sequence similarity and may be considered as orthologous genes and consequently influence the final results. Therefore, other important information across prokaryotic genomes, such as genomic function and genomic structure, should also be considered for orthologous genes identification.

**Figure 2:**
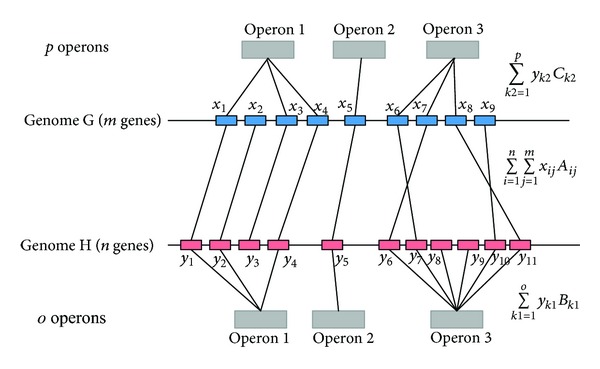
A hypothetical example of orthologous genes between genome G and genome H.

Here we apply a method which is similar to the one proposed by Olman et al. [35] and Mao et al. [36] to determine the orthologous genes between two species. The basic premise is that the orthologous genes between two species should have similar sequence and function and belong to similar operons. The method considers both sequence similarity and other genomic information, such as COG function and operons. So, the orthologous genes identification problem is restated as the problem of maximizing their sequence similarity under the constraint that the orthologous genes are filtered by similar COG function and are grouped into a minimal number of operons. The optimization problem is formulated as follows [36]:

(1) objective function:  ∑i=1n∑j=1mxijAij+∑k1=1oyk1Bk1    +∑k2=1pyk2Ck2,constraint  1:  0≤∑i=1nxij≤1, for  j=1,…,m,   0≤∑j=1mxij≤1, for  i=1,…,n, constraint  2:  ⌈∑i=1lk1∑j=1mxijlk1⌉≤yk1≤1, for  k1=1,…,o,  ⌈∑i=1lk2∑j=1nxijlk2⌉≤yk2≤1, for  k2=1,…,p,

where *m*, *n*, *p*, and *o* represent the number of genes in genome G, genome H, and the number of operons in genome G, genome H, respectively. *x*_*ij*_ represents the existence of an orthology relation between the *j*th gene in genome G and the *i*th gene in genome H, if they are orthologous *x*_*ij*_ = 1, otherwise *x*_*ij*_ = 0. *y*_*k*1_ denotes the *k*1th operon in genome H. If one gene in genome G has an orthologous gene in the *k*1th operon, then *y*_*k*1_ = 1, otherwise *y*_*k*1_ = 0. *y*_*k*2_ denotes the *k*2th operon in genome G. If a gene in genome H is mapped to a gene in the *k*2th operon, then *y*_*k*2_ = 1, otherwise *y*_*k*2_ = 0. *l*_*k*1_  and *l*_*k*2_ represent the number of genes in the *k*1th operon and the *k*1th operon. *A*_*ij*_ is a scaling factor of the score of homologous gene, and *B*_*k*1_ and *C*_*k*2_ are two scaling factors of the reliability of operons. The goal of the optimization problem is to obtain an orthologous relation between the two genomes, namely, an assignment of *x*_*ij*_, *y*_*k*1_, and *y*_*k*2_ that minimizes the objective function. The constraint 1 indicates and guarantees that each gene in genome H can be orthologous to at most one gene in genome G, and each gene in genome G can be orthologous to at most one gene in genome H. The constraint 2 indicates and guarantees that the value of *y*_*k*1_ is 1 while there is any gene in *k*1th operon, which is mapped to at most one gene in genome G, and the value of *y*_*k*2_ is 1 while there is any gene in *k*2th operon, which is orthologous to at most one gene in genome H. The optimization formula guarantees that orthologous gene pairs have maximum sequence and function similarity, and the orthologous genes are grouped into a minimal number of operons. The orthologous genes between genome G and genome H must have similar sequence and function. The sequence similarity is calculated by BLAST, and the function similarity is measured by COG function.  Figure 2 shows the orthologous genes between genome G and genome H in a hypothetical example.

According to the rules in formula (1), we modified the tool PMAP, [36] and the new program can be used to calculate the orthologous genes between two genomes. We calculate orthologous genes for each pair of 617 prokaryotic species using the program and consider those with the *E*-value of BLAST below 10^−3^ as our candidate orthologous genes. Because we think that the orthologous genes are important than operon, the value of *A*_*ij*_, *B*_*k*1_, and *C*_*k*2_ are set to 1.0, 0.5, and 0.5 in the program. In order to reduce the complexity of users, these values could not be modified in the process of using the program.

Table 2 shows the numbers of candidate orthologous genes in five species pairs by different methods. We can see that many special genes are reduced by considering genomic function and genomic structure information. The numbers of orthologous genes between each species pair are used to measure their phylogenetic distance in the next step.

### 2.3. Potential HGT Genes Elimination

After the computation of the numbers of orthologous genes for each pair of 617 prokaryotic species, the phylogenetic distance between each pair of genomes could be measured. However, the putative genes involved in HGT events must be eliminated before calculating the phylogenetic distance. In our work, the genes potentially involved in HGT events will include the highly conserved genes across different species and the genes located on fragments with abnormal genome barcode, which typically correspond to regions of foreign origins [37, 38].

#### 2.3.1. Eliminating the Highly Conserved Orthologous Genes

The highly-conserved genes are those that are orthologous in the overwhelming majority of species. By analyzing the function of the highly conserved genes using COG functional categories [30], we find that most of them belong to the COG code J whose description is “translation, ribosomal structure, and biogenesis.” Most of these genes are considered to be involved in HGT events [39]. The putative HGT genes may have a great influence on the performance of inferring phylogenetic trees, so we should eliminate these genes according to certain criteria. However, for some special set of species, for example, a set containing only species from the same phylum, highly conserved genes are more likely to come from vertical gene transfer events and not from horizontal gene transfer events, so discarding them may cause undesirable results. Here, in order to distinguish these special set of species, we use the following formulas:

(2) $$\begin{array}{c} R_{\text{o}g}\left(\text{G},\text{H}\right)=\frac{2*N_{O}}{N_{\text{G}}+N_{\text{H}}},\\ \text{mean}\;R_{\text{og}}=\frac{2}{n*\left(n-1\right)}\sum_{i=1}^{n-1}\sum_{j=i+1}^{n}R_{\text{og}}\left({\text{G}}_{i},{\text{G}}_{j}\right), \end{array}$$

where *R*_og_(G, H) represents the pair-wise ratio of orthologous genes (ROG) between genome G and genome H and mean *R*_og_ denotes the average pair-wise ROG over a set *S* of *n* species. *N*_O_ is the number of orthologous genes between genome G and genome H, *N*_G_ is the number of genes in genome G, and *N*_H_ is the number of genes in genome H, respectively. G_*i*_ and G_*j*_ represent two genomes in Set *S*. We assume that the closer in phylogenies the two genomes are, the larger their value of ROG. By using mean *R*_og_, we can distinguish the special sets of species and eliminate highly conserved orthologous genes for these sets.

In order to determine the special set of species accurately, we generate different sets of species *S*_1_,…, *S*_*i*_,…, *S*_*m*_ randomly from 617 prokaryotic species for *m* = 10000 times and calculate all the mean *R*_og_(*Si*) values of them. After that, the special sets of species are distinguished by three-sigma rule, which states that for a normal distribution, ordinary values lie within 3 standard deviations of the mean [40]. The formulas are as follows:

(3) $$\begin{array}{c} \mu_{{\text{R}}_{\text{og}}}=\frac{1}{m}\sum_{i=1}^{m}\text{mean}\;R_{\text{og}}\left(S_{i}\right),\\ \sigma_{R_{\text{og}}}={\left(\frac{1}{m-1}\sum_{i=1}^{m}{\left(\text{mean}\;R_{\text{og}}(S_{i})-\mu_{R_{\text{og}}}\right)}^{2}\right),}^{1/2}\\ S_{i}\in \{\begin{array}{cc} \text{oss} & \text{if mean }R_{\text{og}}\left(S_{i}\right)\\ & \in \left[\mu_{R_{\text{og}}}-3\sigma_{R_{\text{og}}},\mu_{R_{\text{og}}}+3\sigma_{R_{\text{og}}}\right],\\ \text{sss} & \text{if mean }R_{\text{og}}\left(S_{i}\right)\\ & \in \left(-\infty ,\mu_{R_{\text{og}}}-3\sigma_{R_{\text{og}}}\right)\cup \left(\mu_{R_{\text{og}}}+3\sigma_{R_{\text{og}}},+\infty \right), \end{array} \end{array}$$

where mean *R*_og_(*S*_*i*_) represents the average ROG of species set *S*_*i*_. oss denotes ordinary species sets and sss denotes special species sets, respectively. The special species sets are those whose species are more similar or more different. For a mixed species set, which have both near and distant species, the species set is defined as an “ordinary” species set.

**Figure 3:**
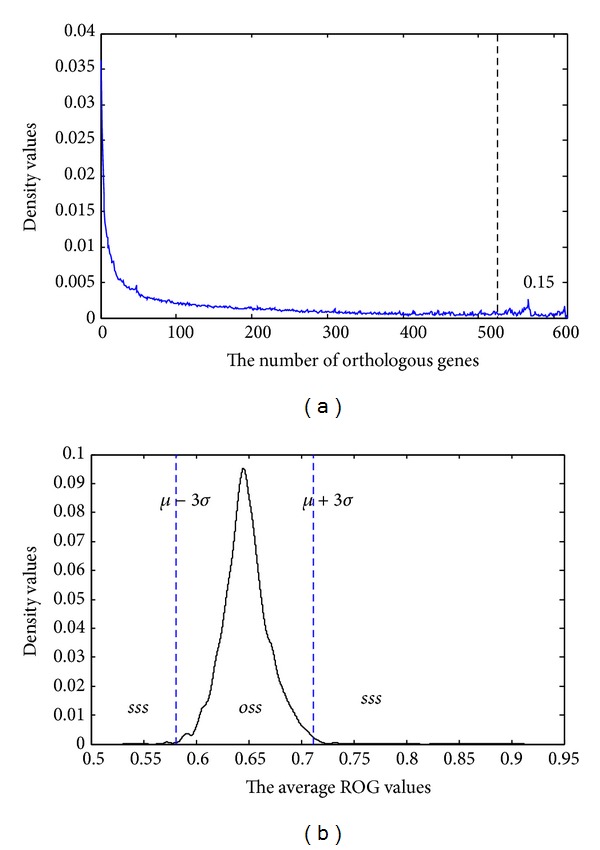
The frequency of (a) different orthologous gene numbers and (b) different average ROG values.

After distinguishing two kinds of set of species, we use different strategies to eliminate the highly conserved genes amang them. The formula is as follows:

(4) $$\begin{array}{c} N_{\text{e}g}=\{\begin{array}{cc} 0.15*N_{\text{as}} & \text{if dataset}\in \text{oss}\\ 0 & \text{if dataset}\in \text{sss}, \end{array} \end{array}$$

where *N*_as_ represents the number of all species (*N*_as_ = 617 in our run). *N*_eg_ denotes the number of eliminations among the highly conserved orthologous genes. The eliminated genes are obtained by their rank of similarity in other genomes. The threshold is chosen by the frequency of different orthologous gene numbers on the 617 species.  Figure 3(a) illustrates the frequency of different orthologous gene numbers on the 617 species. From Figure 3(a), we can see that with the increase in the number of orthologous genes, the density values are getting smaller until the number of orthologous gene is near 525 (617∗0.85). The special distribution of the highly conserved orthologous genes may be related to special events of biological evolution, such as HGT. So, in this paper, we select 0.15 (1–0.85) as the threshold.  Figure 3(b) illustrates the density values of different average ROG values on 10000 random species sets. From Figure 3(b), it can be seen that the average ROG values on most datasets distribute between 0.6 and 0.7. By using the three-sigma rule, the ordinary set of species and the special set of species are neatly distinguished.

#### 2.3.2. Eliminating Abnormal Genome Barcode Genes

Barcodes of genomes describe the distribution of the combined frequency of each *k*-mer and its reverse complement at each fragments of *L* bps across sequences of a genome, whole or in part [37]. Most regions of barcodes in one genome have high similarity, while a few of them are different and usually are considered to be abnormal fragments. The abnormal fragments may be generated by horizontal gene transfers (HGT) or phage invasions during biological evolution.

**Figure 4:**
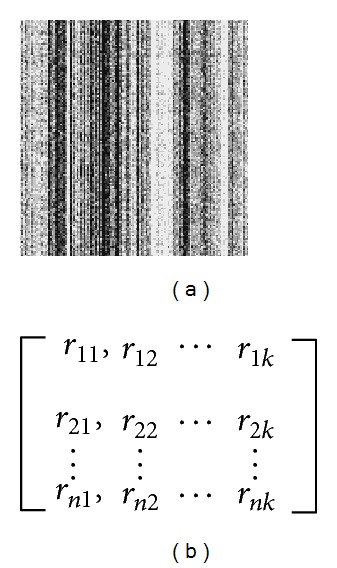
A hypothetical example: (a) barcode figure and (b) matrix structure of the genomic barcode.

In our work, each genome is partitioned into nonoverlapping fragments of 1000 bps (*L* = 1000) long and represented by a 4-mer-based barcode. We use *M* to represent the length of the genome sequence. Each fragment of one genome is represented as a vector of 136 values, and each genome is represented as a matrix with *N* (*N* = ⌊*M*/*L*⌋) rows and 136 columns.  Figure 4 shows the barcode figure and the matrix of a hypothetical genome.  Figure 4(a) illustrates the barcode figure of the hypothetical genome, and Figure 4(b) illustrates the matrix structure of the genomic barcode. Each element *r*_*ij*_ is the frequency of the *j*th  4-mer in the *i*th DNA fragment. Each row of the matrix means the barcode for each genomic fragment. Consequently, each column represents the frequencies of a specific 4-mer, such as “AATG” or “GTCG” over the different DNA fragments, and each row represents the frequencies of the different 4-mer over a specific DNA fragment.

The average barcode of the whole genome is a row vector whose elements are the mean values of each column of the barcode across all fragments. The distances between the average barcode and the barcode for each genomic fragment, namely, the row of the barcode corresponding to the fragment taken as a row vector, are calculated by Euclidean distance between the two 136-dimensional row vectors. The formulas are as follows:

(5) avgBarcode(k) =1N∑i=1Nbarcode(i,k) (N=⌊M/L⌋,  k=1,...,136),disBarcode(i)=∑k=1136(barcode(i,k)−avgBarcode(k))2Rge(x)=m(disBarcode(i)) +3∗s(disBarcode(i)) (i=1,…,N),std Rge=s(Rge(j)) (j=1,…,|S|),

where avgBarcode(*k*) represents the *k*th element of the average barcode of the whole genome and disBarcode(*i*) denotes the distance between the average barcode and the barcode of the *i*th fragment. *N* is the number of fragments in the genome. *R*_ge_(*x*) represents the value of genomic evolution in genome X, and std *R*_ge_ denotes the standard deviation of genomic evolution ratio in a set of species. *m*() and *s*() are the mean value and the standard deviation, and |*S*| is the number of species in a dataset *S* for inferring phylogenies. We assume that the closer in phylogenies the genome X and genome Y are, the more similar their values of genomic evolution. In our current study, we eliminate only the abnormal genome barcode genes for the set of species which have high standard deviation of the genomic evolution value. So by using std*R*_ge_, we can distinguish the special set of species and eliminate the abnormal genome barcode genes fome these datasets.

In order to evaluate the special species sets accurately, we generate different species sets 10000 times randomly and calculate all the std *R*_ge_ of them. After that, the special species sets are distinguished by three-sigma rule, which states that for a normal distribution, ordinary values lie within 3 standard deviations of the mean [40]. The formulas are defined as follows:

(6) $$\begin{array}{c} \mu_{R_{g\text{e}}}=\frac{1}{m}\sum_{j=1}^{m}\text{std}\;R_{g\text{e}}\left(S_{j}\right),\\ \sigma_{R_{g\text{e}}}={\left(\frac{1}{m-1}\sum_{j=1}^{m}{\left(\text{std}{\;R}_{g\text{e}}\left(S_{j}\right)-\mu_{R_{g\text{e}}}\right)}^{2}\right),}^{1/2}\\ S_{j}\in \{\begin{array}{cc} \text{oss} & \;\text{if}\;\text{std}\;R_{g\text{e}}\left(S_{j}\right)\in \left(-\infty ,\mu_{R_{g\text{e}}}+3\sigma_{R_{g\text{e}}}\right]\\ \text{sss}\; & \text{if}\;\text{std}\;R_{g\text{e}}\left(S_{j}\right)\in \left(\mu_{R_{g\text{e}}}+3\sigma_{R_{g\text{e}}},+\infty \right), \end{array} \end{array}$$

where std *R*_ge_(*S*_*j*_) represents the standard deviation of genomic evolution ratio in species set *S*_*j*_ and *m* is the number of species sets. In our work, *m* is equal to 10000. oss denotes ordinary species sets, and sss denotes special species sets, respectively.

After distinguishing two kinds of species sets, we use different strategies to eliminate the abnormal genome barcode genes from them. There are about 20% genes of a genome belonging to potential genes which involve HGT event [41], so the formula is defined as follows:

(7) $$\begin{array}{c} N_{\text{e}g}\left(j\right)=\{\begin{array}{cc} 0 & \text{if}\;\text{dataset}\in \text{oss}\\ 0.2*N_{g}\left(j\right) & \text{if}\;\text{dataset}\in \text{sss}\\ & \left(j=1,\ldots ,\left|S\right|\right), \end{array} \end{array}$$

where *N*_*g*_(*j*) represents the number of genes in species *j* and |*S*| is the number of species in the dataset for inferring phylogenies. *N*_eg_(*j*) denotes the number of eliminated abnormal genome barcode genes.

**Figure 5:**
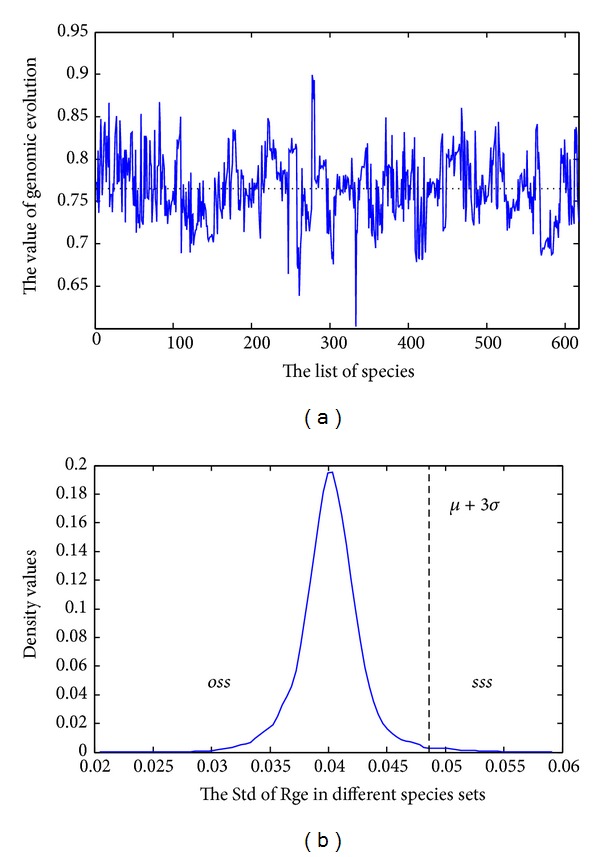
(a) The ratios of genomic evolution on 617 species and (b) the density of different standard deviation of *R*_ge_.

Figure 5(a) illustrates the values of genomic evolution on 617 species. From Figure 5(a), we can see that the ratios of genomic evolution are different in different species, the maximum genomic evolution ratio is approximately equal to 0.9, and the minimum genomic evolution ratio is approximately equal to 0.6.  Figure 5(b) illustrates the density values of different standard deviation of *R*_ge_(*x*) on 10000 random species sets. From Figure 5(b), it can be seen that the standard deviation of *R*_ge_(*x*) on most datasets distributes between 0.035 and 0.045. By using three-sigma rule, the ordinary and special sets of species are neatly distinguishable.

### 2.4. Phylogenetic Distance Measurement

In this section, we introduce a novel phylogenetic distance between a pair of genomes that is based on the orthologous genes obtained in Section 2.3. As a consequence, the distance takes indirectly into account sequence similarity and other genomic information. The conserved gene cluster distance (CGCD) measures the conserved extent of orthologous genes between two genomes. CGCD is the log inverse of the number of orthologous genes that are in the conserved clusters. The conserved gene cluster distance is defined as follows:

(8) $$\begin{array}{c} D_{\text{c}g\text{c}}\left(\text{X},\text{Y}\right)=-\log\;\left(N_{\text{c}g\text{c}}\right), \end{array}$$

where *N*_cgc_ is the total number of orthologous genes in conserved clusters between genome X and genome Y.

**Figure 6:**
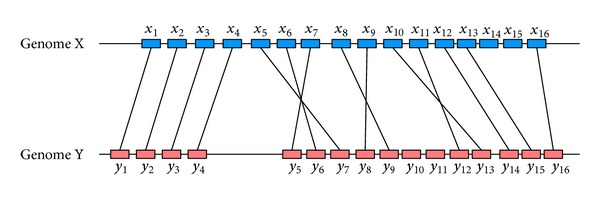
Conserved orthologous gene clusters between pairs of genomes.

Figure 6 shows an example of the conserved orthologous gene clusters between the hypothetical genome X and genome Y. To calculate the phylogenetic distance between two genomes, the conserved orthologous gene clusters need first to be determined. In Figure 6, there are 16 genes in both genome X and genome Y, 14 orthologous genes between two genomes, and four conserved orthologous gene clusters. The gene clusters *x*_1_ *x*_2_ *x*_3_ *x*_4_ and *y*_1_ *y*_2_ *y*_3_ *y*_4_  compose an orthologous gene cluster with conserved arrangement. In this case, the order of the genes of the cluster in genome X is the same in genome Y. The gene clusters  *x*_5_ *x*_6_ *x*_7_ and *y*_5_ *y*_6_ *y*_7_  compose an orthologous gene cluster with inverted arrangement. In this case, the order of the genes in the cluster in genome X is inverted in genome Y. The gene clusters *x*_8_ *x*_9_ *x*_10_ *x*_11_, *y*_8_ *y*_9_ *y*_12_ *y*_13_, and *x*_12_ *x*_13_ *x*_16_, *y*_14_ *y*_15_ *y*_16_ are examples of insertion and deletion in conserved orthologous gene clusters. We consider all the above cases as conserved orthologous gene cluster. In the example shown in Figure 6, *N*_cgc_ is 14 and *D*_cgc_ is −1.1461.

We validated the stability of the conserved orthologous gene clusters in biological evolution by using the genome's barcodes [37] for the corresponding sequences. The distance of two species in phylogenetic tree is reported to be related with the similarity in genomic sequence, functions, and structures [5]. The number of conserved orthologous gene clusters between two genomes indicates the conserved orthologs of them in biological evolution. By using conserved orthologous gene clusters, the method considers less gene rearrangement, insertion, and deletion. Due to their high stability in biological evolution, the conserved orthologous gene clusters should be able to represent the evolutionary relationship precisely.

**Figure 7:**
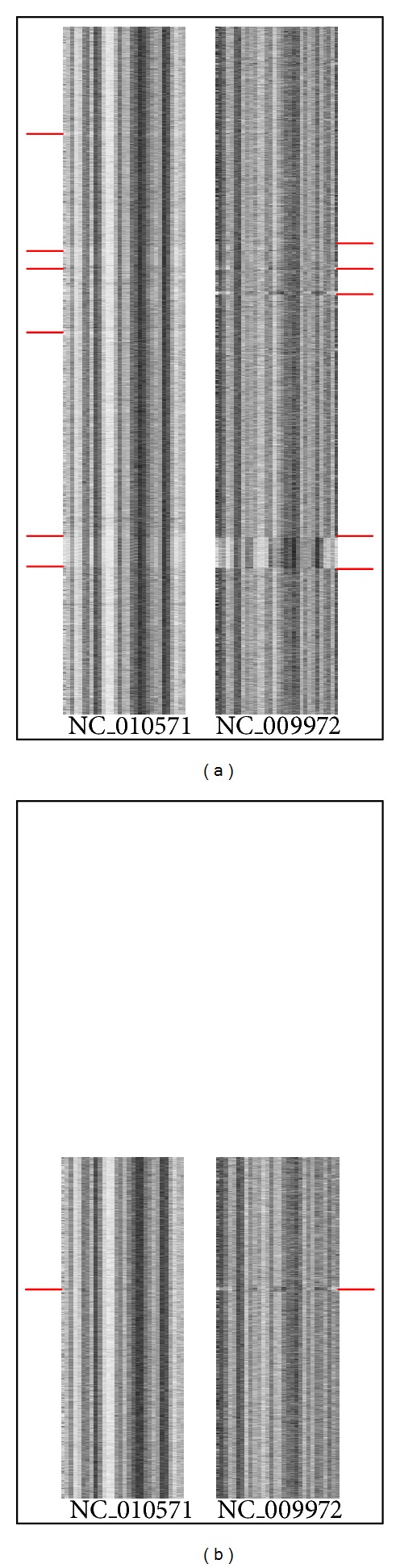
The barcodes of sequences from (a) orthologous genes and (b) orthologous genes of conserved clusters.

Figure 7 illustrates the barcodes of sequences from orthologous genes and orthologous genes of conserved clusters between NC_010571 and NC_009972 as an example.  Figure 7(a) shows the barcodes of sequences generated from orthologous genes by the method in Section 2.2. From Figure 7(a) we can see that there are many abnormal fragments which are not intrinsic genes but arguably originated from another organism. These fragments would influence the performance of inferring phylogenies.  Figure 7(b) illustrates the barcodes of sequence generated from the genes in conserved orthologous gene clusters found by the above method. From Figure 7(b), it can be seen that there are few abnormal fragments in sequences of two species. Therefore, the conserved orthologous gene clusters could be more effective for inferring phylogenies than the original orthologous genes.

### 2.5. Phylogenies Construction

The phylogenetic distance matrix is calculated from the numbers of orthologous genes in the conserved clusters by the CGCD formula defined above. The result is an *m* × *m* distance matrix where *m* is the number of species in the set used for the inference. In order to construct the phylogenetic tree, we apply the neighbor-joining (NJ) method [27, 28] on the distance matrix. The neighbor-joining (NJ) method which has been implemented availably in the tool “Phylip” (available at: http://www.phylip.com/) was initially proposed by Saitou and Nei [27] to reconstruct phylogenetic trees from evolutionary distance data. In this paper, the principle of this method is to find pairs of operational taxonomic units that minimize the total branch length at each stage of clustering. By using distance data, the method can construct an unrooted or rooted tree to simulate the phylogenetic tree. The package includes several programs with implementation of different phylogenies inferring algorithms on different kinds of data and utilities for carrying out phylogenies-related tasks. We used the Neighbor-Joining (NJ) method to construct the phylogenic tree and the program “Drawgram” to plot the rooted phylogenic tree.

### 2.6. Performance Measurement

One of the challenges of inferring phylogenies is how to measure the inferring performance of a method. Most of the previous phylogenetic analysis methods measured the performance with respect to an organism taxonomy. However, there is no uniform standard prokaryotic organism taxonomy [5]. In this paper, we use Bergey's taxonomy to measure the quality of the results of our method. Bergey's taxonomy is a classification scheme in Bergey's Manual of Systematic Bacteriology [29], which has been widely recognized by most biologists as the reference taxonomy. In order to facilitate result comparison between the inferred phylogenetic tree and the tree from the Bergey's taxonomy, the number of agreed quartet topologies is used to measure the performance. The quartet topology model was proposed by Lin et al. [5], and it is a subtree structure of the subset of four terminal taxa which are connected in three possible ways. The quartet topologies of Bergey's taxonomy are generated from the classifications of the 617 prokaryotic genomes described in Section 2.1. There are 2,684,646,031 effective quartets of the 617 prokaryotic genomes.

## 3. Results and Discussion

In order to facilitate the comparison of the results, the proposed method is initially examined on the set of species used to evaluate ComPhy [5]. Then we examined the proposed method on the datasets of the 617 prokaryotic genomes of different species of archaea and bacteria. To validate the method completely, we also apply the method on random sets of species with different dimensions. The agreed quartet topologies between the inferred phylogenetic tree and the tree from the Bergey's taxonomy are used to measure the performance of the method, and the whole accuracy of all 617 prokaryotic genomes is 91.12% on effective quartets.

### 3.1. Fixed Datasets Validation

In this validation step, the method is examined using the datasets applied to ComPhy. These are 9 datasets of different dimensions, 7 datasets were formed randomly from datasets of archaea and bacteria, and the other two were obtained from BPhyOG [18] and Deeds et al. [19]. All of these datasets are subsets of the 617 prokaryotic genomes.

We run CGCPhy and other methods on these 9 datasets. The performance results on the 9 datasets of GCD [16], OG [18], CVTree [21–23], ComPhy [5], and CGCPhy are shown in Table 3. From the table, we can see that the accuracies of CGCPhy on most datasets are higher than those of other methods except for dataset 4, in which the performance of CGCPhy is a little lower than the ComPhy method. The accuracies of the 9 datasets by GCD, OG, CVTree, ComPhy, and CGCPhy are 86.28% ± 0.0399, 84.99% ± 0.0268, 92.86% ± 0.0390, 93.76% ± 0.0339, and 96.98% ± 0.0193, respectively. The method CGCPhy presents the highest average accuracy and the lowest standard deviation, outperforming the other four methods of phylogenies inference.

**Figure 8:**
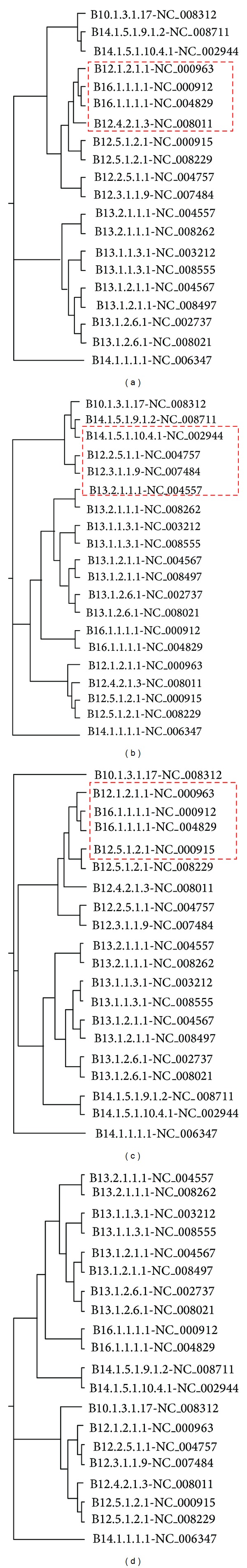
Phylogenetic trees of 20 bacterial species by (a) GCD, (b) CVTree, (c) ComPhy and (d) CGCPhy.

To further investigate the effectiveness of each method, we draw by different methods the phylogenetic trees of 20 bacterial species selected randomly which are a subset of the dataset 1 in Table 1.  Figure 8 shows four phylogenetic trees inferred by the GCD, CVTree, ComPhy, and CGCPhy methods. These phylogenetic trees are plotted by the program “drawgram” in the tool “Phylip.” Figure 8(a) illustrates the phylogenetic tree inferred by gene content distance (GCD) method. From Figure 8(a), we can see that the genomes of NC_000912 and NC_004829, which are members of Firmicutes phylum (B13), are placed outside their own phylum and inferred in Proteobacteria phylum (B12).  Figure 8(b) illustrates the phylogenetic tree inferred by composition vector tree (CVTree) method. From Figure 8(b), it can be seen that the genomes of NC_004757 and NC_007484, which are members of protebacteria phylum (B12), are placed outside their own phylum and inferred near Actinobacteria phylum (B14).  Figure 8(c) illustrates the phylogenetic tree inferred by composite distance (ComPhy) method. From Figure 8(c), we can see that the genomes of NC_000912 and NC_004829, which are members of Firmicutes phylum (B13), are placed outside their own phylum and inferred in protebacteria phylum (B12).  Figure 8(d) illustrates the phylogenetic tree inferred by conserved gene cluster distance (CGCPhy) method. From Figure 8(d), it can be seen that all genomes are accurately placed closer to their own phylum.

In order to estimate the contribution of each different step in the phylogenetic distance computation, we use different combinations of the three stages: (1) identifying orthologous genes, (2) eliminating the highly conserved orthologous genes, and (3) measuring conserved gene cluster distance to infer phylogenetic tree.  Table 4 shows the performance results of 4 different combinations of the three stages on the 9 datasets. From the table, it can be seen that accuracies on all the datasets applying all the three stages combination are higher than the others. The average accuracies of the 9 datasets by combinations 1, 1 + 2, 1 + 3, and 1 + 2 + 3 are 83.41%, 83.97%, 95.43%, and 96.98%, respectively. The results provide clear evidence that each step plays a relevant role for inferring phylogenetic tree. It can be seen that the performances of the methods are improved smaller by considering the two stages of removing highly conserved genes and abnormal genome barcode genes than considering the stage of gene clusters. The reason may be that the role of orthologous genes is more important than HGT genes in the evolutionary process. However, in several datasets, such as dataset 1, dataset 2 and dataset 6, the performances are improved greatly by considering the two stages. So, we keep step two in our approach for processing several special datasets.

**Figure 9:**
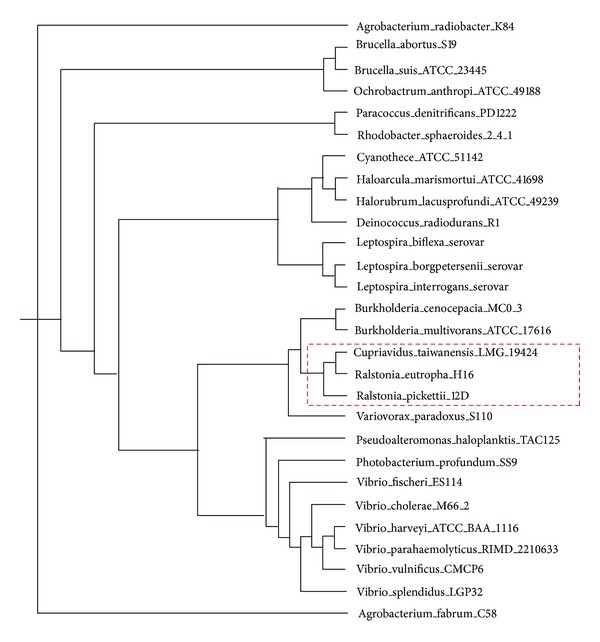
Phylogenetic trees of 28 multiple-chromosome prokaryotic genomes by CGCPhy.

We also construct the phylogenetic tree by CGCPhy on 28 multiple-chromosome prokaryotic genomes, and the results are shown in Figure 9. From Figure 9, it can be seen that most species in the same genus are closer in the phylogenetic tree. However, *Ralstonia eutropha* in *Ralstonia* are closer with *Cupriavidus taiwanensis* in *Cupriavidus* than *Ralstonia pickettii* in the same genus. The result may be that we calculate orthologous gene clusters between two genomes but not between different chromosomes on multiple-chromosome prokaryotic species.

### 3.2. Random Datasets Validation

In this validation, the methods are examined using random sets of species with dimension 50, 100, 200, and 300, respectively. For each dimension, we randomly generate 10 subsets of the set of the 617 prokaryotic genomes.

**Figure 10:**
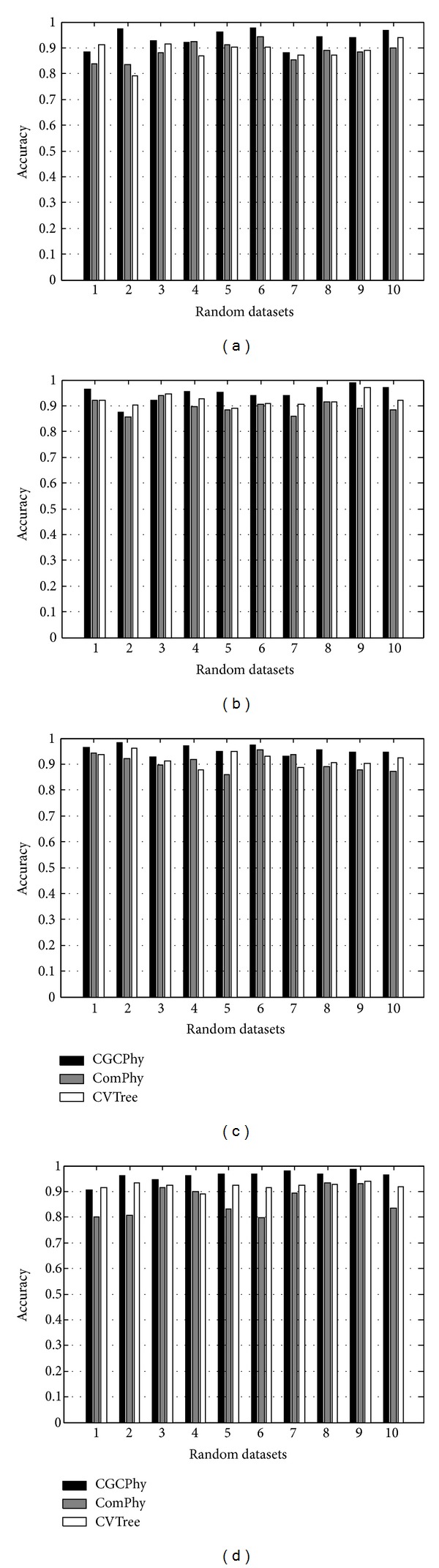
The accuracy of phylogenetic inference in (a) 50, (b) 100, (c) 200, and (d) 300 random species datasets.

The accuracy results on random species sets of different dimension by CVTree [21–23], ComPhy [5], and CGCPhy are illustrated in Figure 10. Figures 10(a)–10(d) illustrate the accuracy of phylogenetic inference by the same methods in sets of 50, 100, 200, and 300 random species, respectively. From these figures, we can see that the accuracy on most datasets of CGCPhy is higher than that of CVTree and ComPhy. The average accuracy on random datasets of 50 species of CVTree, ComPhy, and CGCPhy are 88.63%, 88.60%, and 93.74%, respectively. The average accuracy on random datasets of 100 species of CVTree, ComPhy, and CGCPhy are 92.09%, 89.55%, and 94.81%, respectively. The average accuracy in random datasets of 200 species of CVTree, ComPhy, and CGCPhy are 91.87%, 90.71%, and 95.48%, respectively. The average accuracy on random datasets of 300 species of CVTree, ComPhy, and CGCPhy are 92.20%, 86.49%, and 96.13%, respectively. The method CGCPhy has higher average accuracy than CVTree and ComPhy for all the dimensions of the random species datasets. The performance results validate the effectiveness of the method for phylogenetic inference.

The performance results on the random sets of different combinations of the three stages of the distance computation are illustrated in Table 5. From the table, we can see that the accuracies in 50, 100, 200, and 300 random species datasets are 78.51% ± 0.0389, 81.04% ± 0.0564, 93.65% ± 0.0279, and 95.04% ± 0.0278, respectively. The method CGCPhy has higher average accuracy and lower standard deviation in different datasets, so it has excellent stability and accuracy in phylogenetic inference. The method proposed in this paper achieved average above 93% agreement with Bergey's taxonomy in quartet topologies on random datasets of different species number. Empirical results show that the proposed method has excellent qualities for phylogenetic analysis.

We also examine three special kinds of species sets by using CGCPhy. Firstly, we used species from the same family and order as the first kind of species sets. Then the phylogenetic trees are constructed by CGCPhy, and the average accuracies are 93.48% and 89.04% agreement with Bergey's taxonomy. Secondly, we generated 10 species sets by the following criteria: a set with only distant species. We used CGCPhy to construct the phylogenetic trees, and the average accuracies are 88.76%. Because of the small number of orthologous genes between distant species, the accuracies are not as good as expected. We also used 10 random sets which contain species from distant genera and similar genera. By using CGCPhy, the average accuracy of constructed phylogenetic trees is 92.70%.

Prokaryotic phylogenies inferring is still an open problem, and Bergey's taxonomy changes the taxonomy of several species in each new release [42]. For example, the genus *Oceanobacillus* was part of the phylum Proteobacteria (B12) in Bergey's taxonomy 3.0 [43], but it belonged to phylum Firmicutes (B13) in Bergey's taxonomy 5.0 [29]. The species *Oceanobacillus iheyensis* (B13.1.1.1.12) is accurately placed to its own phylum by our CGCPhy method. In Bergey's taxonomy 5.0 [29] the species *Thiomicrospira denitrificans* was placed in phylum Gammaproteobacteria (B12.3). But by CGCPhy the species of *Thiomicrospira* are placed closer to phylum Epsilonproteobacteria (B12.5), and in TOBA 7.7 [44] the species was renamed *Sulfurimonas denitrificans* and moved to Epsilonproteobacteria. The species *Carboxydothermus hydrogenoformans* (NC_007503) and *Moorella thermoacetica* (NC_007644) are placed closer by using CGCPhy. But in Bergey's taxonomy 5.0, they were placed in Order Clostridiales (B13.1.1) and Order Thermoanaerobacteriales (B13.1.2). However, in Bergey's Manual Vol. 3 [32], NC_007503 were moved in Order Thermoanaerobacterales (B13.2.3). So, the proposed method has important implications in phylogenetic inference and has a powerful capability for phylogenetic analysis.

## 4. Conclusions

How to infer prokaryotic phylogenies accurately is still an open problem. In this paper, we infer prokaryotic phylogenies by a novel method CGCPhy, which is based on the distance matrix of orthologous gene clusters between whole genome pairs. Unlike most of existing methods, which only use several genome information, our method measures the evolutionary relationship among various groups of species by using genomic information of sequence, function, and structure. If two species are closer in phylogenetic tree, they are more similar in genomic sequence, functions, and structures. So, we measure orthologous genes by both considering sequence similarity and other genomic annotation information. Nonetheless, these orthologous genes may contain several genes which are involved in horizontal gene transfer (HGT) events. In this paper, we eliminate the highly conserved genes across different species and the genes located on the fragments with abnormal genome barcode which are considered as the putative genes potentially involved in HGT events. Afterwards, the distance of orthologous gene cluster between two genomes is calculated by the number of orthologous genes in conserved clusters. Using these distances, we can construct phylogenetic trees by the third-party tool “Phylip.”

The proposed method is examined on different datasets from 617 prokaryotic genomes. To validate the method completely, we examine the method on the fixed datasets and random datasets in different number of species. The method achieved average above 93% agreement with Bergey's taxonomy in quartet topologies on these datasets. The CGCPhy method has higher average accuracy of phylogenetic inference than other methods, such as CVTree and ComPhy in most datasets. Simultaneously, it has low standard deviation of inferring accuracy in different datasets. Simulation results show that the proposed method has consistent robustness and accuracy in phylogenetic inference and has a potential capability for phylogenetic analysis. The complied program and test datasets are publicly available at http://csbl.bmb.uga.edu/publications/materials/weidu/.

## Figures and Tables

**Table 1:** Taxon statistics of the 645 prokaryotic genomes.

Phylum	Class	Order	Family	Genus	Species	Strain
Archaea1	1	3	5	10	15	15
Archaea2	9	10	15	23	31	34
Archaea3	1	1	1	1	1	1
Bacteria1	1	1	1	1	1	1
Bacteria2	1	1	1	4	6	6
Bacteria4	1	2	2	2	3	4
Bacteria6	2	3	3	4	6	6
Bacteria10	2	5	5	11	16	32
Bacteria11	1	1	1	2	5	5
Bacteria12	6	35	58	125	227	307
Bacteria13	3	7	18	32	90	143
Bacteria14	3	11	18	21	42	53
Bacteria15	1	1	1	1	1	1
Bacteria16	1	1	2	3	9	13
Bacteria17	1	1	2	3	7	8
Bacteria19	2	2	2	2	2	2
Bacteria20	3	3	5	8	11	12
Bacteria21	1	1	1	1	1	1
Bacteria22	1	1	1	1	1	1

Total 19	41	90	142	255	475	645

**Table 2:** The candidate orthologous genes in five species pairs.

Compared genomes pair	B	B + C	B + C + O
NC_000916 versus NC_007716	88	86	73
NC_007681 versus NC_008025	300	296	258
NC_008228 versus NC_007356	557	515	469
NC_003155 versus NC_002678	2249	1524	1437
NC_002655 versus NC_002695	4693	4617	4221

B: bi Directional best hits BLAST; C: COG; O: operon.

**Table 3:** The performance results of accuracy by five methods.

DS	NoS	GCD^∗(%)^	OG^∗(%)^	CT^(%)^	Com^∗(%)^	CGC^(%)^
1	52	86.44	83.93	92.17	90.29	**98.83**
2	53	86.40	85.49	87.92	90.74	**95.29**
3	54	87.34	88.27	91.47	96.55	**98.91**
4	82	92.58	84.35	**98.87**	96.16	96.59
5	96	85.45	87.22	99.24	99.26	**99.29**
6	165	77.98	87.87	89.86	94.38	**98.04**
7	181	89.74	80.34	90.08	95.67	**96.35**
8	277	84.04	81.89	93.88	90.71	**96.00**
9	398	86.56	85.52	92.28	90.07	**93.56**

DS: datasets; NoS: number of species; GCD: gene content distance; OG: overlapping gene distance; CT: CVTree, *K* = 5; Com: ComPhy; CGC: CGCPhy. *The results of GCD, OG, and ComPhy obtained from reference [5].

**Table 4:** The results of accuracy by different stage combinations on fixed datasets.

Datasets	1	1 + 2	1 + 3	CGCPhy
Dataset1	84.67%	87.08%	95.52%	**98.83%**
Dataset2	87.01%	89.58%	90.58%	**95.29%**
Dataset3	91.92%	86.46%	96.95%	**98.91%**
Dataset4	87.53%	86.58%	95.73%	**96.59%**
Dataset5	84.96%	84.96%	**99.29%**	**99.29%**
Dataset6	78.80%	78.96%	95.76%	**98.04%**
Dataset7	78.11%	78.11%	**96.35%**	**96.35%**
Dataset8	77.48%	77.48%	**96.00%**	**96.00%**
Dataset9	80.26%	86.54%	92.72%	**93.56%**

Mean	83.41%	83.97%	95.43%	**96.98%**

1: identifying orthologous genes; 2: eliminating the highly conserved orthologous genes; 3: measuring conserved gene cluster distance; CGCPhy = 1 + 2 + 3.

**Table 5:** The results of accuracy by different stage combinations on random datasets.

Species no.	1	1 + 2	1 + 3	CGCPhy
50	77.62%	78.56%	93.74%	**93.74%**
100	77.78%	80.04%	93.37%	**94.81%**
200	79.67%	81.41%	94.08%	**95.49%**
300	78.97%	84.16%	93.39%	**96.13%**

Mean	78.51%	81.04%	93.65%	**95.04%**
Std	0.0389	0.0564	0.0279	**0.0278**

1: identifying orthologous genes; 2: eliminating potential HGT genes; 3: measuring conserved gene cluster distance; CGCPhy = 1 + 2 + 3.
